# Long-term survival in advanced pulmonary large-cell neuroendocrine carcinoma after multi-line immunochemotherapy and anti-angiogenic therapy: a case report

**DOI:** 10.3389/fimmu.2025.1618672

**Published:** 2025-06-30

**Authors:** Hongming Wang, Shiyan Li, Xiaoyang Liu, Yan Zhang, Jiang Zheng, Yangfeng Du, Zemin Xiao, Nuoni Wang, Zhijun Wu

**Affiliations:** ^1^ Department of Oncology, Changde Hospital, Xiangya School of Medicine, Central South University (The First People’s Hospital of Changde City), Changde, China; ^2^ Department of Electrocardiogram and Physiology, Changde Hospital, Xiangya School of Medicine, Central South University (The First People’s Hospital of Changde City), Changde, China

**Keywords:** immunochemotherapy, anti-angiogenic therapy, survival, pulmonary large-cell neuroendocrine carcinoma, case report

## Abstract

**Background:**

Pulmonary large-cell neuroendocrine carcinoma (PLCNEC) is a rare and highly aggressive subtype of lung cancer with neuroendocrine features, typically diagnosed at an advanced stage. Its clinical presentation and treatment response resemble those of small cell lung cancer, whereas its histological characteristics are more similar to those of non-small cell lung cancer. The rarity and heterogeneity of PLCNEC have impeded the development of standardized treatment protocols. Conventional approaches such as surgery, chemotherapy, and radiotherapy alone have yielded poor outcomes, underscoring the need for more effective therapeutic strategies.

**Case description:**

This report presents the case of a 45-year-old Chinese woman with advanced PLCNEC who received first-line treatment with a four-drug regimen consisting of etoposide, cisplatin, camrelizumab, and endostar. This was followed by maintenance therapy with camrelizumab and endostar, local palliative radiotherapy, re-administration of etoposide and cisplatin, and hepatic artery interventional embolization. Notably, her progression-free survival (PFS) after first-line therapy (i.e., PFS1) reached 2 years. Second-line therapy with atezolizumab, bevacizumab, and docetaxel achieved a PFS2 of 1 year. Third-line treatment maintained atezolizumab and introduced the anti-angiogenic agent anlotinib and chemotherapeutic agent pemetrexed, resulting in a PFS3 of 6 months. The patient tolerated all treatments well, with no grade 3 or 4 adverse events observed, and achieved a total overall survival of 47 months.

**Conclusions:**

This case illustrates the potential for long-term survival in advanced PLCNEC through intensive, multi-line combination therapies. The favorable outcome observed in this patient supports further investigation of such combination strategies in clinical studies.

## 1 Introduction

Lung cancer, the leading cause of cancer-related mortality, comprises two main subtypes: small cell lung cancer (SCLC) and non-small cell lung cancer (NSCLC). SCLC accounts for approximately 15% of all lung cancers and is highly aggressive, with a 5-year survival rate of only 7%. In contrast, NSCLC represents approximately 85% of cases and has a comparatively higher 5-year survival rate of approximately 25% (1). Pulmonary large cell neuroendocrine carcinoma (PLCNEC) is a high-grade neuroendocrine tumor most commonly seen in geriatric male smokers, accounting for approximately 3% of primary lung tumors. A retrospective analysis of 103,890 SCLC and 3,303 LCNEC cases demonstrated superior prognosis in LCNEC versus SCLC: median overall survival (OS) was 10 versus 7 months, with 5-year survival rates of 17% and 5%, respectively (2). Over half of patients with LCNEC present with distant metastases at initial diagnosis—predominantly involving the brain, bone, liver, and lungs—with poor prognosis; median OS is approximately 5 months, with a 9% 2-year OS rate (3). Large cell neuroendocrine carcinoma (LCNEC) is challenging to diagnose pathologically and is defined by neuroendocrine morphology (such as organoid nesting with palisading, trabecular architecture, and rosette formations), cytological features of non-small cell carcinoma (including large cells, prominent nucleoli, and/or abundant cytoplasm), and a high proliferation rate. As LCNEC tumors are poorly differentiated, immunohistochemical labeling is often essential for diagnosis. In the absence of definitive neuroendocrine morphology, the diagnosis requires the expression of at least one of three standard neuroendocrine markers: synaptophysin, chromogranin A, or neural cell adhesion molecule (4, 5).

The molecular classification of PLCNEC holds significant clinical and research value. However, a precise classification has yet to be established, largely owing to the limited number of comprehensive studies in this area. Rekhtman et al. demonstrated the molecular heterogeneity of PLCNEC through retrospective next-generation sequencing (NGS), identifying three distinct subtypes: SCLC-like, NSCLC-like, and carcinoid-like forms. These subtypes are defined by specific genetic mutations and are associated with varying chemotherapy sensitivities and survival outcomes. Further investigation into the molecular characteristics of LCNEC is essential to inform and refine treatment strategies (6). The optimal management of LCNEC remains uncertain, as most available data come from small-sample, retrospective studies, and phase III or randomized clinical trials are lacking.

This report presents a case of advanced PLCNEC in a patient whose tumor was reclassified as NSCLC-like LCNEC through NGS, and whose comprehensive treatment plan incorporated chemotherapeutic, immunotherapeutic, and anti-angiogenic agents commonly used for SCLC and NSCLC. This approach resulted in a prolonged OS of nearly 4 years while maintaining a high quality of life during treatment. These encouraging outcomes support the potential of combination therapy strategies in PLCNEC management.

## 2 Case presentation

A 45-year-old Chinese woman presented with intermittent coughing of white sputum, left-sided chest pain, fatigue, loss of appetite, and exertional shortness of breath since November 4, 2020. Although she appeared weak, she exhibited no fever, chills, or other notable symptoms and had an Eastern Cooperative Oncology Group performance status of 2. She had no history of smoking or alcohol use and no family history of tumors or genetic disorders. On physical examination, palpation revealed an enlarged left supraclavicular lymph node measuring approximately 1.0 × 1.5 cm and hepatomegaly, with the liver edge palpable three transverse fingers below the costal margin. No other significant physical findings were noted.

**Figure 1:**
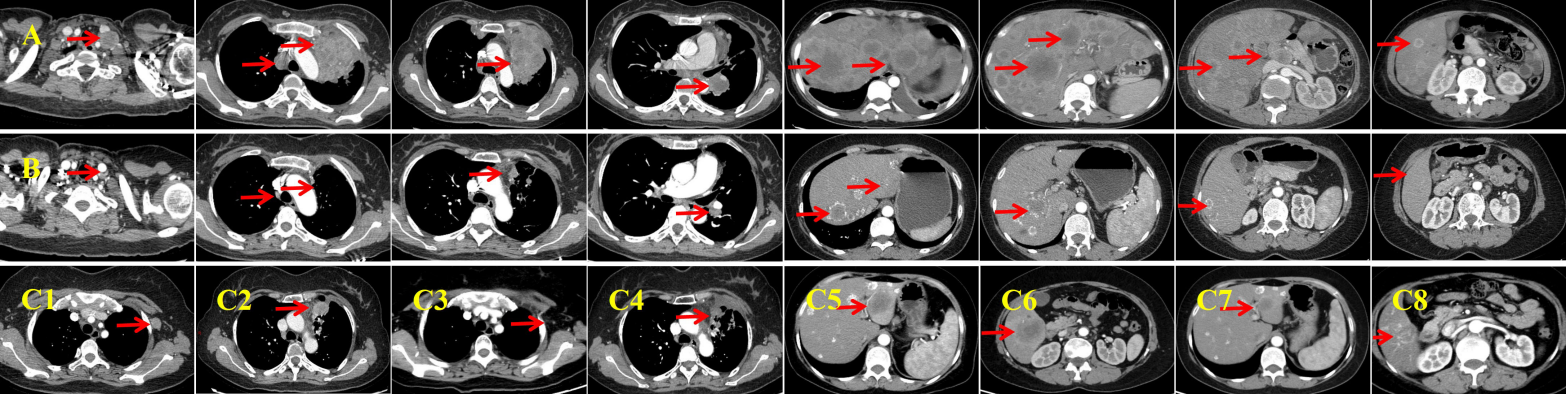
Baseline imaging and first-line therapy assessments. **(A)** Baseline contrast-enhanced computed tomography (CT) performed on January 14, 2021 reveals a left lung mass measuring approximately 55 × 80 mm, with associated lymphadenopathy and liver metastases. The lesions exhibit marked central necrosis and heterogeneous enhancement. **(B)** After six cycles of first-line therapy, an enhanced CT on July 29, 2021 demonstrates significant shrinkage of all lesions and the appearance of calcification in the liver metastases. **(C, C1, C2)** Follow-up enhanced CT on October 26, 2021 indicates progression of the primary lung lesion and the emergence of a new, enlarged lymph node in the left axilla. **(C3, C4)** Post-radiotherapy enhanced CT (January 22, 2022) shows shrinkage of the primary lesion and near-complete resolution of the left axillary lymph node. The response is classified as a partial response (PR). **(C5, C6)** Enhanced CT performed on July 28, 2022 reveals new metastatic lesions in the liver, indicating progressive disease. **(C7, C8)** Following three cycles of resumed first-line systemic therapy combined with local interventional treatment, an enhanced CT on November 2, 2022 shows significant reduction of liver metastases. The imaging response is again classified as PR.

**Figure 2:**
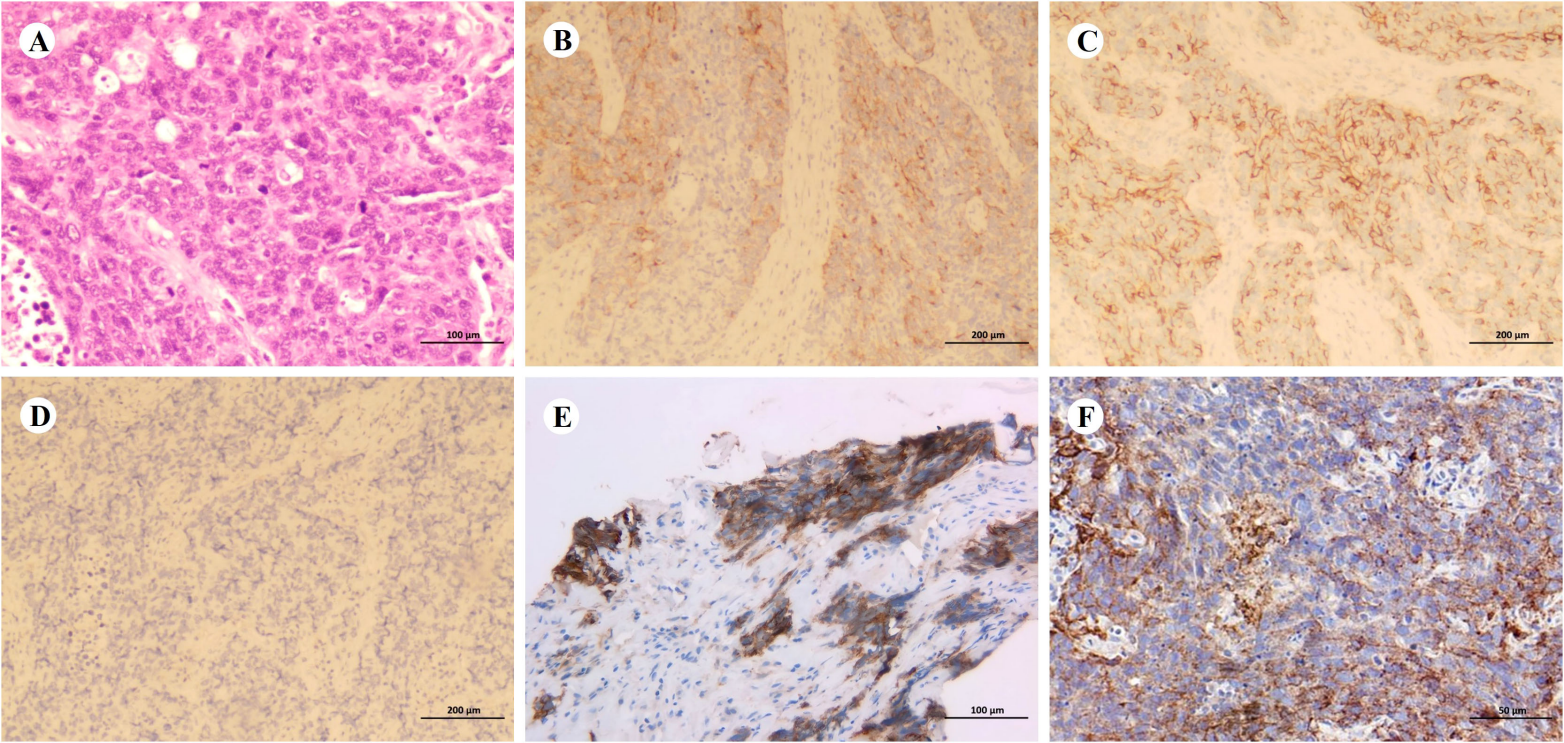
Pathological diagnosis. **(A–D)** Representative hematoxylin and eosin staining (**A**, ×200; scale bar = 100 µm) and immunohistochemical staining (**B**–**D**, ×100; scale bar = 200 µm) of the biopsy specimen. Tumor cells are positive for synaptophysin **(B)** and neural cell adhesion molecule **(C)**, but negative for chromogranin A **(D)**. **(E, F)** Programmed death-ligand 1 (PD-L1) expression before treatment (**E**, ×200; scale bar = 100 µm; tumor proportion score [TPS]: 50%) and during treatment (**F**, ×400; scale bar = 50 µm; TPS: 5%).

Baseline imaging and first-line therapy assessments are shown in **Figures 1A–C**. Contrast-enhanced computed tomography (CT) performed on January 14, 2021 revealed multiple lesions in the left lung and liver, along with multiple enlarged lymph nodes (**Figure 1A**). Pathological findings are detailed in **Figures 2A–F**. CT-guided biopsy of the left lung mass confirmed the diagnosis of LCNEC (**Figures 2A–D**). Contrast-enhanced magnetic resonance imaging of the brain showed no evidence of metastatic lesions, whereas a bone scan revealed metastases in the L2 vertebra, right acetabulum, left femoral head, and left ischium. The disease was staged as cT4N3M1c, stage IVB, according to the eighth edition of the International Association for the Study of Lung Cancer staging system. NGS did not identify actionable driver mutations but did detect alterations in AKT1, MAP2K1, and TP53, along with high programmed cell death ligand 1 (PD-L1) expression (tumor proportion score [TPS]: 50%; **Figure 2E**). Based on the absence of RB1+TP53 co-mutations, the tumor was molecularly reclassified as NSCLC-like LCNEC (7).

A four-drug regimen comprising camrelizumab (200 mg, Day 1), endostar (210 mg, continuous intravenous infusion for 72 h), etoposide (100 mg/m^2^, Days 1–3), and cisplatin (75 mg/m^2^, Day 1) was initiated as first-line therapy. The patient developed grade I–II gastrointestinal toxicities and grade I myelosuppression during chemotherapy. Imaging assessment indicated a partial response (PR) after six treatment cycles (**Figure 1B**). She then received maintenance therapy with camrelizumab and endostar for 10 cycles between July 30, 2021 and June 24, 2022. During this period, palliative radiotherapy was initiated on October 30, 2021 in view of the local progression of the primary lesion in the left upper lung and the emergence of a new, enlarged lymph node in the left axilla (**Figures 1C1, C2**). The planning gross tumor volume dose was 60.20 Gy, delivered in 28 fractions. During chemotherapy-free intervals, the patient exhibited a high quality of life, evidenced by an Eastern Cooperative Oncology Group (ECOG) performance status of 0.

**Figure 3:**
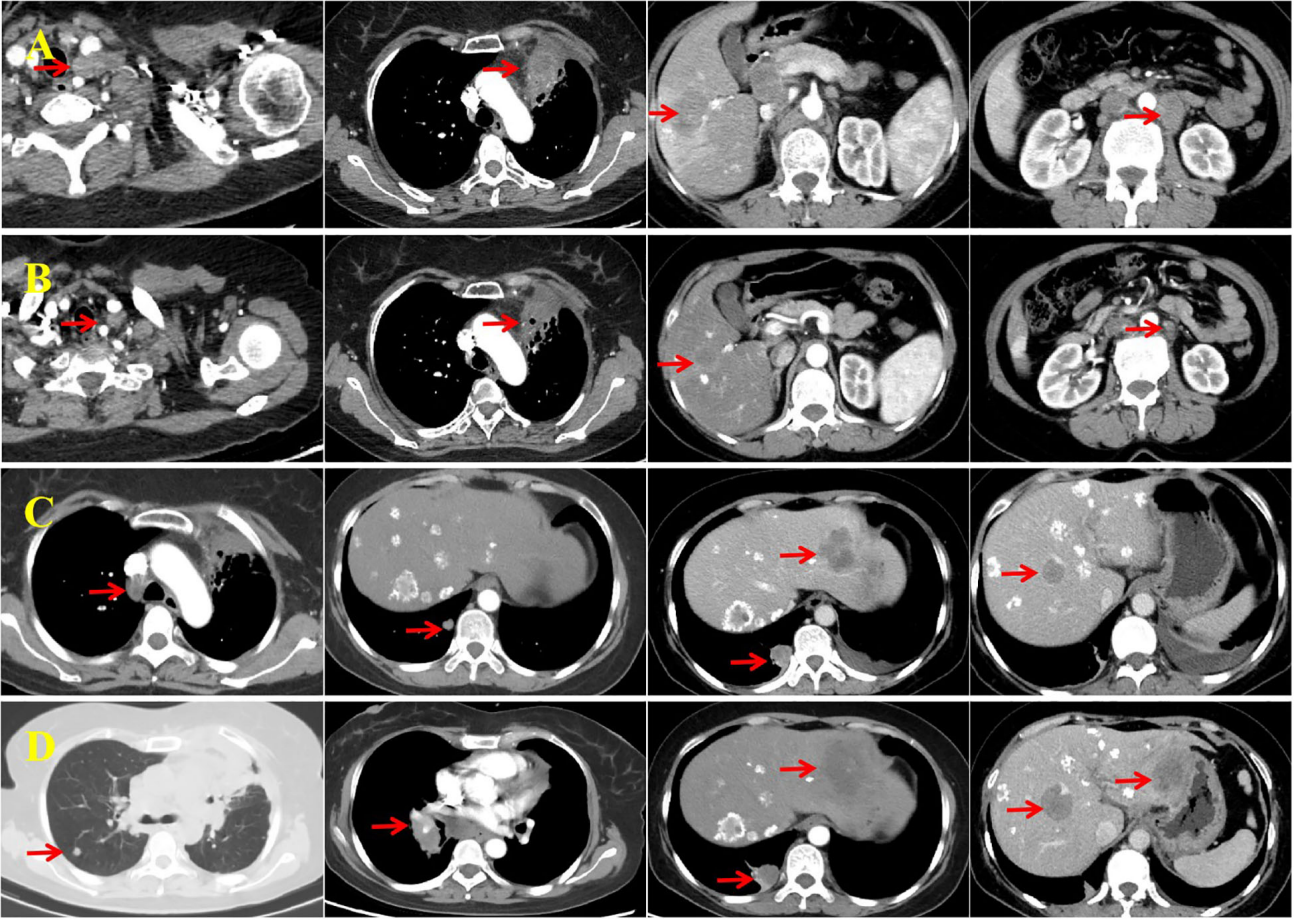
Imaging assessments conducted before and after second- and third-line therapy. **(A)** Enhanced computed tomography (CT) performed on February 10, 2023 shows progression of the left supraclavicular fossa lymph node and primary lung lesion, along with new liver metastases and enlarged retroperitoneal lymph nodes. **(B)** After four cycles of second-line therapy, enhanced CT (June 31, 2023) reveals a reduction in the size of all lesions, with the response assessed as a partial response (PR). **(C)** Enhanced CT on February 14, 2024 shows enlarged mediastinal lymph nodes and new metastases in the lungs and liver, consistent with progressive disease (PD). **(D)** The most recent CT scan (August 20, 2024) shows further enlargement of mediastinal lymph nodes, increased size and number of bilateral lung metastases, and progression of liver metastases. The imaging confirms PD.

Radiotherapy yielded a PR in the localized lesions (**Figures 1C3, C4**). However, enhanced CT imaging on July 28, 2022 revealed progression of the liver lesions compared with previous scans (**Figures 1C5, C6**). Therefore, the initial four-drug regimen was resumed, along with hepatic artery interventional embolization. Intraoperative hepatic artery perfusion chemotherapy with cisplatin (60 mg) was also administered. A PR was observed after three cycles of the resumed regimen (**Figures 1C7, C8**). Despite this, disease progression was evident after six cycles (**Figure 3A**), prompting a switch to second-line therapy. The new regimen included immune rechallenge with atezolizumab (1,200 mg, Day 1), combined with bevacizumab (15 mg/kg, Day 1) as anti-angiogenic therapy, and single-agent docetaxel (75 mg/m^2^, Day 1) as chemotherapy.

After completing four cycles of second-line therapy, imaging on June 31, 2023 indicated a PR (**Figure 3B**). For second-line maintenance treatment, a three-drug combination regimen—including chemotherapy—was continued, and the patient received a total of 12 cycles. However, an imaging evaluation on February 14, 2024 showed disease progression (**Figure 3C**). A rebiopsy of the left supraclavicular fossa lymph node confirmed LCNEC, and repeat NGS did not detect any driver mutations but revealed a TP53 mutation and decreased PD-L1 expression (TPS: 5%; **Figure 2F**).

**Figure 4:**
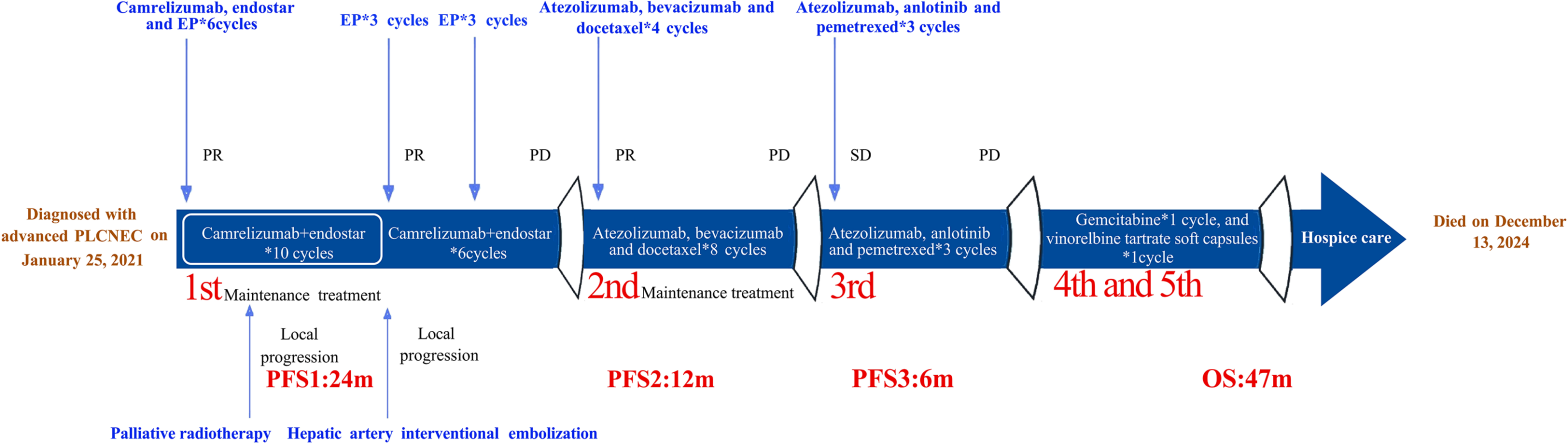
Patient treatment course.

Considering the availability of complimentary atezolizumab, immunotherapy was continued across treatment lines, in combination with pemetrexed (500 mg/m^2^, Day 1) and anti-angiogenic therapy using anlotinib (10 mg orally, Days 1–14). After three cycles of this third-line regimen, imaging indicated stable disease. However, progressive disease was observed 6 months later (**Figure 3D**) and the patient’s ECOG performance status was 2. She did not receive further immunotherapy or anti-angiogenic therapy thereafter. The patient subsequently received one cycle each of gemcitabine (1,000 mg/m^2^, Days 1 and 8) and vinorelbine tartrate soft capsules (60 mg/m^2^, Days 1 and 8). She did not tolerate further additional chemotherapy owing to medical concerns and was transitioned to hospice care at a local hospital, where she died on December 13, 2024. Her treatment course is illustrated in **Figure 4**.

## 3 Discussion

A standardized treatment protocol for LCNEC has not yet been established. For patients with surgically resectable stage I–III LCNEC, radical surgical resection should be performed as early as possible (8). However, LCNEC is an aggressive tumor with a high recurrence rate, even after complete resection. Therefore, adjuvant radiotherapy and chemotherapy are recommended to reduce recurrence and improve survival (9). For patients with stage IV LCNEC, chemotherapy is generally more beneficial than other treatment options in view of the neuroendocrine tumor characteristics (10). SCLC chemotherapy regimens are most commonly used, including etoposide plus platinum-based agents (cisplatin or carboplatin) (3). In contrast, one study reported that patients with LCNEC who received first-line NSCLC chemotherapy regimens—such as gemcitabine or paclitaxel combined with platinum—had longer OS than those treated with etoposide plus platinum (11).

Consensus is lacking on whether LCNEC should be treated using NSCLC or SCLC chemotherapy protocols. However, advances in genomic and transcriptomic profiling have begun to reveal associations between LCNEC subtypes and differential chemotherapy responses, providing insights that may inform the development of personalized treatment approaches. In a retrospective study, patients with SCLC-like LCNEC receiving an etoposide-platinum doublet SCLC regimen achieved a significantly better disease control rate (100% vs 20%, P = 0.007), response rate (75% vs 0%, P = 0.02), and median progression-free survival (mPFS; 8.3 vs 2.4 months, P = 0.002) than those on a pemetrexed-platinum doublet NSCLC regimen; differences in OS were not significant (9.7 vs 4.1 months, P = 0.6) (7). For patients with NSCLC-like LCNEC, etoposide-platinum demonstrated non-inferior or improved PFS and OS versus NSCLC regimens, indicating that it is a potentially optimal therapeutic strategy for most LCNEC cases. However, comprehensive genomic subtyping revealed pemetrexed-platinum as the superior regimen in NSCLC-like LCNEC, supporting expanded NGS panel implementation to identify patients benefitting from regimen-specific therapy. The optimal frontline chemotherapy remains controversial, with cisplatin-etoposide preference persisting irrespective of SCLC-like or non-SCLC-like classification. These inconsistencies, predominantly derived from retrospective small-cohort analyses, highlight the need for prospective trials to establish evidence-based guidelines. Another retrospective study by Derks et al. which included 79 patients with LCNEC treated with platinum-based doublet chemotherapy, classified patients based on RB1 mutation status (RB1-mutant vs RB1-wild-type). Among RB1-wild-type patients, those treated with a NSCLC regimen (platinum plus gemcitabine or paclitaxel) had significantly better PFS and OS compared with those treated with a SCLC regimen (platinum plus etoposide): PFS was 6.1 vs 5.7 months (P = 0.019) and OS was 9.6 vs 5.8 months (P = 0.026). In contrast, among RB1-mutant patients, differences in PFS and OS between chemotherapy regimens were not significant (12).

Immune checkpoint inhibitors (ICIs) are now the standard first-line therapeutic agents for advanced NSCLC and SCLC, significantly improving survival and prognosis (13). Although imperfect as a biomarker, PD-L1 expression in tumor and/or immune cells remains the logical predictor for PD-1/PD-L1 inhibitor response (14). However, owing to the rarity of LCNEC, studies investigating the use of ICIs in this population remain limited. LCNEC exhibits high PD-L1 expression and a median tumor mutation burden of 5.42 mutations per megabase, suggesting that immunotherapy may be effective in this setting (15–17). Fisch et al. retrospectively analyzed 191 patients with LCNEC, demonstrating a median OS of 26.4 months with ICI therapy versus 9.0 months for other first-line platinum-based doublets and 4.0 months for non-platinum-based chemotherapies (18). Shirasawa et al. evaluated 70 advanced LCNEC cases, revealing significantly longer OS in anti-PD-1-treated patients compared to untreated patients (25.2 vs 10.9 months) (19). In our patient, initial PD-L1 expression was high (TPS: 50%) but declined substantially to a low level (TPS: 5%) in the rebiopsy specimens. Although this marked reduction conventionally suggests potential immunotherapy resistance, the patient sustained significant clinical benefit from combination therapy including immunotherapy, as evidenced by her OS.

Furthermore, Evangelou et al. conducted a non-randomized, prospective study (the LANCE study) evaluating the efficacy of atezolizumab in combination with chemotherapy in patients with stage IV LCNEC (20). The objective response rate was 50% in the atezolizumab plus chemotherapy group, compared with that of the chemotherapy-only group at 42.9%. After a median follow-up of 23.3 months, the immunochemotherapy group had not yet reached median PFS or OS, whereas the chemotherapy-only group had a median PFS of 5.2 months and median OS of 8.2 months. These findings provide prospective evidence supporting the survival benefit of combining atezolizumab with platinum-based chemotherapy in patients with metastatic LCNEC.

LCNEC and SCLC are both high-grade neuroendocrine tumors that share similar clinical characteristics, including high malignancy, rapid progression, and poor prognosis. Although progress has been made in first-line treatment for advanced LCNEC and SCLC, further optimization remains essential. Recently, combined anti-angiogenic therapy has emerged as a promising research area, with potential to enhance treatment efficacy and delay tumor progression by targeting tumor angiogenesis. Tumor vascular abnormalities (e.g., tortuosity, leakage, and low pericyte coverage) result in hypoxia and impaired infiltration of immune cells. Anti-angiogenic drugs (e.g., bevacizumab) inhibit angiogenesis and alleviate the immunosuppressive microenvironment by blocking vascular endothelial growth factor (VEGF) signaling. Immunotherapy can also modulate vascular remodeling, thereby establishing a cycle of immunostimulation and vascular remodeling within tumors.

Combined antiangiogenic and immunotherapeutic strategies exert synergistic effects via multiple mechanisms (21). Preclinical evidence indicates that anlotinib monotherapy improves the tumor immune microenvironment through PD-L1 downregulation, thereby inhibiting tumor progression (22). In murine models of lung cancer, combining anlotinib with a PD-1 inhibitor enhanced innate immune cell infiltration and yielded synergistic antitumor effects (23). A four-drug combination regimen that incorporates ICIs and anti-angiogenic agents has shown substantial therapeutic advances in extensive-stage SCLC. The phase III randomized controlled ETER701 study demonstrated that the anti-angiogenic agent anlotinib, when combined with the PD-L1 inhibitor benmelstobart and chemotherapy regimen of etoposide plus carboplatin, significantly prolonged median PFS to 6.9 months and median OS to 19.3 months in patients with extensive-stage SCLC (24). Similarly, a prospective study evaluating a combination of surufatinib, toripalimab, etoposide, and cisplatin reported a median PFS of 6.9 months and median OS of 21.1 months in advanced SCLC, the longest survival duration achieved with medical therapy in this setting to date (25). Although the second interim analysis of the BEAT-SC trial did not show a survival benefit with bevacizumab + atezolizumab + cisplatin/carboplatin + etoposide compared with that of placebo + atezolizumab + cisplatin/carboplatin + etoposide, the addition of bevacizumab did improve investigator-assessed PFS (5.7 vs 4.4 months, P = 0.006) (26). Across these studies, the overall side effect profiles of the four-drug regimens were manageable, and no new safety signals were observed.

Endostar, a novel recombinant human endostatin, mediates its anti-angiogenic activity primarily via the VEGF-associated signaling pathway. Preclinical research revealed that combining endostar with an anti-PD-1 antibody yielded potent synergistic effects, suppressing Lewis lung carcinoma growth through tumor microenvironment amelioration and autophagy induction (27). A clinical case demonstrated successful first-line treatment of advanced LCNEC using endostar plus pembrolizumab and platinum-based chemotherapy (28). This regimen showed favorable tolerability with PFS exceeding 24 months, representing an effective and safe first-line therapeutic approach for advanced LCNEC. In China, it is commonly used in the treatment of advanced NSCLC. Our patient was initially diagnosed with advanced NSCLC-like LCNEC, characterized by a high tumor burden and rapid progression. A chemotherapy regimen commonly used for SCLC, namely etoposide and cisplatin, was selected and combined with camrelizumab and endostar to form a four-drug intensive regimen as first-line therapy. This combination was associated with mild adverse effects and yielded a favorable therapeutic outcome. Notably, the liver metastases gradually shrank and developed calcification, an uncommon phenomenon in clinical practice.

PD-1 and PD-L1 inhibitors function within the same signaling pathway. Their binding interrupts PD-1/PD-L1 interaction, restoring immune cell cytotoxic function against tumor cells and counteracting immune escape (29). However, PD-1 and PD-L1 inhibitors differ in antibody architecture, target engagement, efficacy, and immune-related toxicities (30, 31). Most PD-1 inhibitors are IgG4 antibodies, targeting mainly immune T cells, while PD-L1 inhibitors are mostly IgG1 antibodies, targeting predominantly tumor cells (32). Compared to PD-1 inhibitors, PD-L1 inhibitors demonstrate a lower incidence of grade ≥ 3 adverse events, potentially attributable to the retention of the PD-L2 pathway by PD-L1-targeted agents. This preserved pathway may facilitate local homeostasis of macrophage PD-L2 signaling, thereby mitigating immune hyperactivation-associated toxicities (33).

Although programmed cell death protein-1 (PD-1) and PD-L1 inhibitors both target immune checkpoints, their mechanisms of anti-tumor activity are not entirely congruent, and the variability in their efficacy in advanced LCNEC remains poorly understood (34, 35). A small retrospective study suggested that switching between PD-1 and PD-L1 inhibitors may be an effective treatment strategy for certain patients with advanced NSCLC (36). Another retrospective study found that patients with advanced NSCLC who initially responded to ICIs maintained clinical benefit when rechallenged with subsequent ICI therapy (37). A case of advanced driver gene-negative NSCLC achieved imaging-confirmed complete response following anti-PD-L1 antibody (atezolizumab) therapy after progression on anti-PD-1 (nivolumab) treatment (38). This suggests some differences in the mechanism of action between PD-1 and PD-L1 inhibitors that may affect the pattern of resistance. PD-L1 inhibitors have a greater blocking capacity and may overcome resistance to PD-1 inhibitors, especially in patients with an active CD80 pathway, because over-expression of CD-80 is one of the mechanisms of resistance to PD-1 inhibitors (39). Another case report demonstrated a PR to anti-PD-L1 (atezolizumab) following primary resistance to prior anti-PD-1 (nivolumab) therapy. Therefore, ICI rechallenge (e.g., switching to a different target drug) may be effective in some patients and should be combined with treatment duration and biomarkers (e.g., PD-L1 expression) for population screening. Since current evidence is mostly derived from retrospective studies or case reports, prospective studies are required to validate sequential treatment strategies (40).

We employed a triple combination of atezolizumab, bevacizumab, and docetaxel as second-line therapy following disease progression on a first-line PD-1 inhibitor-based combination regimen. This approach resulted in a PFS of up to 1 year, highlighting the feasibility of immune rechallenge in combination with anti-angiogenic and chemotherapeutic agents. A multicenter retrospective study similarly concluded that continuing immunotherapy as part of second-line treatment after disease progression may improve survival outcomes in patients with advanced NSCLC (41). Subsequently, owing to the availability of free atezolizumab through a grant program, the patient received a third-line crossover regimen comprising atezolizumab, pemetrexed, and anlotinib, which achieved a median PFS of up to 6 months. Although the OS of our patient reached 47 months and individual contributions of immunotherapy, chemotherapy, and anti-angiogenic agents remain difficult to isolate, the fact that combination regimens incorporating all three modalities contributed 42 months to disease control underscores their therapeutic significance. These findings suggest that combination strategies intensify treatment and extend the window for subsequent-line therapies, collectively contributing to a significant improvement in survival.

Data on targeted therapies for LCNEC remain limited. However, recent reports have described cases of patients with LCNEC harboring actionable driver mutations such as epidermal growth factor receptor mutations, anaplastic lymphoma kinase rearrangements, rearranged during transfection fusions, and B-Raf proto-oncogene serine/threonine kinase V600E mutations that have shown responsiveness to tyrosine kinase inhibitors (42–45). These findings highlight the importance of genetic testing in patients with LCNEC. In our patient, despite two biopsies and NGS, no driver mutations were detected, and she was therefore ineligible for targeted therapy. Delta-like ligand 3, a ligand in the Notch signaling pathway, is frequently expressed in SCLC and LCNEC, making it a promising therapeutic target (46). Rovalpituzumab tesirine, a humanized monoclonal antibody targeting delta-like ligand 3, demonstrated notable antitumor activity in a phase I clinical trial involving patients with high-grade neuroendocrine tumors (74 with SCLC and 8 with LCNEC), with 11 of 60 evaluable patients (18%) achieving a confirmed objective response (47). Additional potential targets, such as neurotrophic receptor tyrosine kinase, mechanistic target of rapamycin, and poly ADP-ribose polymerase 1, are currently in the early stages of investigation and warrant further research (48–50).

In conclusion, immunochemotherapy combined with anti-angiogenic therapy represents a promising treatment approach for patients with advanced PLCNEC, with the potential to improve survival outcomes. However, the use of such combination regimens should be guided by careful assessment of biomarker status, tumor burden, and patient tolerability. Further clinical trials are needed to validate their efficacy and safety.

## Patient perspective

Before passing, the patient expressed satisfaction with the treatment regimen and its effectiveness. She demonstrated a clear understanding of the disease process and was fully informed about the potential adverse effects of combination therapy strategies.

## Data Availability

The original contributions presented in the study are included in the article/supplementary material. Further inquiries can be directed to the corresponding authors.
